# MALDI-TOF MS Analysis of Condensed Tannins with Potent Antioxidant Activity from the Leaf, Stem Bark and Root Bark of *Acacia confusa*

**DOI:** 10.3390/molecules15064369

**Published:** 2010-06-15

**Authors:** Shu-Dong Wei, Hai-Chao Zhou, Yi-Ming Lin, Meng-Meng Liao, Wei-Ming Chai

**Affiliations:** Key Laboratory of the Ministry of Education for Coastal and Wetland Ecosystems, School of Life Sciences, Xiamen University, Xiamen 361005, China

**Keywords:** *Acacia confusa*, condensed tannins, MALDI-TOF MS, antioxidant activity

## Abstract

The structures of the condensed tannins from leaf, stem bark and root bark of *Acacia confusa* were characterized by matrix-assisted laser desorption/ionization time-of-flight mass spectrometry (MALDI-TOF MS) analysis, and their antioxidant activities were measured using 1,1-diphenyl-2-picrylhydrazyl (DPPH) free radical scavenging and ferric reducing/antioxidant power (FRAP) assays. The results showed that the condensed tannins from stem bark and root bark include propelargonidin and procyanidin, and the leaf condensed tannins include propelargonidin, procyanidin and prodelphinidin, all with the procyanidin dominating. The condensed tannins had different polymer chain lengths, varying from trimers to undecamers for leaf and root bark and to dodecamers for stem bark. The condensed tannins extracted from the leaf, stem bark and root bark all showed a very good DPPH radical scavenging activity and ferric reducing power.

## 1. Introduction

**Figure 1:**
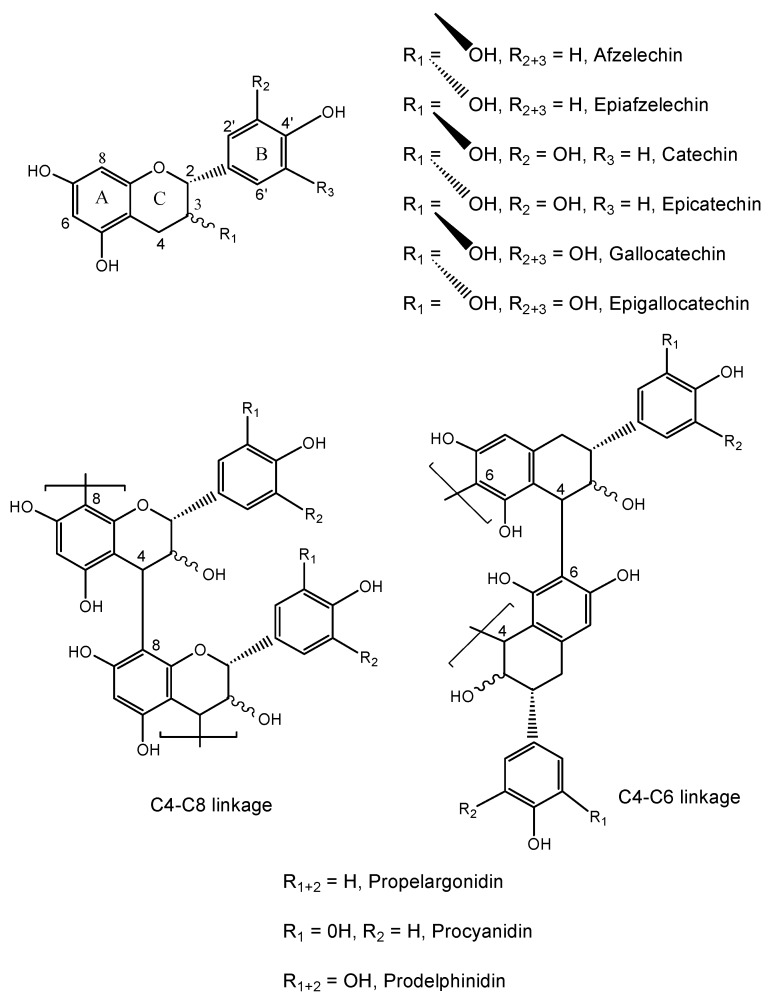
Chemical structure of ﬂavan-3-ol monomer units and condensed tannins.

Tannins are polyphenols that occur in plants, where they can amount to 20% of the plant dry weight, depending on the plant and organ [1,2,3]. Two types of tannins occur in vascular plants: the condensed and the hydrolysable [4]. Condensed tannins are formed of flavan-3-ol units, which are linked together through C4–C6 or C4–C8 bonds to oligomers and high molecular weight polymers [5,6,7,8]. The diversity of condensed tannins is given by the structural variability of the monomer units: different hydroxylation patterns of the aromatic rings A and B, different stereochemistry at the chiral centers C2 and C3, and the distinct location and stereochemistry of the interflavanoid bond (Figure 1). 

Condensed tannins are considered as functional ingredients in botanical, nutritional supplements and therefore they are attracting more attention. However, the bioactivity capacity of plant condensed tannins is generally recognized to be largely dependent on their structure and particularly the degree of polymerization [9,10]. The structural elucidation of these compounds, especially the higher polymers, is difficult because of their heterogeneous character. Due to the complexity and diversity, the characterization of highly polymerized condensed tannins thus remains very challenging, and less is known regarding structure-activity relationships [11,12]. Various techniques including NMR, acid-catalyzed depolymerization of the polymers in the presence of nucleophilic reagents, and MALDI-TOF MS have been used to characterize condensed tannins [13,14,15,16,17,18,19].

*Acacia confusa* is traditionally used as a medicinal plant [20]. An aqueous extract of *A. confusa* leaves was used in Taiwan for wound healing and anti-blood-stasis [21]. The crude extracts of heartwood, leaf, and bark contain a wide variety of phenolic compounds [20,22,23,24,25,26] and some also show an excellent antioxidant activity [22,25]. Therefore, this plant might be a good candidate for further development as a nutraceutical or an antioxidant remedy. Previous studies showed that the structures of the main monomers constituting the condensed tannins from leaves, twigs, and branches of *A. confusa* were catechin and epicatechin [27,28]. However, detailed information on the condensed tannins’ profiles, including polymer chain length, chemical constitution of individual chains, and the sequential succession of monomer units in individual chains, has not been reported. In this study, contents of total phenolics and extractable condensed tannins of leaf, stem bark and root bark of *A. confusa* were determined, and the structures of condensed tannins from them were characterized by MALDI-TOF MS. In addition, the free radical scavenging capacities and ferric reducing power of condensed tannins from leaf, stem bark and root bark are also discussed.

## 2. Results and Discussion

### 2.1. Content of total phenolics and extractable condensed tannins

Root bark had the highest contents of total phenolics and extractable condensed tannins, followed by stem bark and leaf (Table 1). Plant phenolics constitute one of the major groups of compounds acting as primary antioxidants or free radical terminators [29]. Phenolic compounds are considered to be the major contributor to the antioxidant activity of vegetables, fruits or medicinal plants. The antioxidant activities of phenolic compounds are attributed to their redox properties, which allow them to act as reducing agents, hydrogen donators, singlet oxygen quenchers, *etc*. [22,30]. Our results revealed that *A. confusa* (especially root bark and stem bark) had high levels of phenolics, and might be potential sources of natural antioxidants.

**Table 1:** Contents of total phenolics and extractable condensed tannins in leaf, stem bark and root bark of *A. confusa*.

Samples	Total phenolics (mg/g )	Extractable condensed tannins (mg/g)
Leaf	180.08 ± 2.67c	64.17 ± 1.44c
Stem bark	394.69 ± 5.03b	247.76 ± 10.93b
Root bark	467.99 ± 6.22a	280.70 ± 11.75a

Using respective purified tannins from leaf, stem bark and root bark as the standards. Different letters in the same column show significant differences from each other at P < 0.05 level.

### 2.2. MALDI-TOF MS analysis

MALDI-TOF MS is very sensitive to molecular weight, and nowadays is considered a method of choice for analysis of tannins exhibiting large structural heterogeneity. With this technique fragmentation of the analyte molecules upon laser irradiation can be substantially reduced by embedding them in a light absorbing matrix. As a result, intact analyte molecules are desorbed and ionized along with the matrix, and they can be analyzed in a mass spectrometer [31,32]. MALDI-TOF MS produces only a singly charged molecular ion for each parent molecule and allows detection of high mass with precision [33]. Several factors including the selection of an appropriate matrix, optimal mixing and optimal selection of cationization reagent must be optimized to develop MALDI-TOF MS techniques. When Cs^+^ was employed as the cationization reagent for MALDI, Chinese gallotannins gave a relatively simple MALDI-TOF spectrum [34].

**Figure 2:**
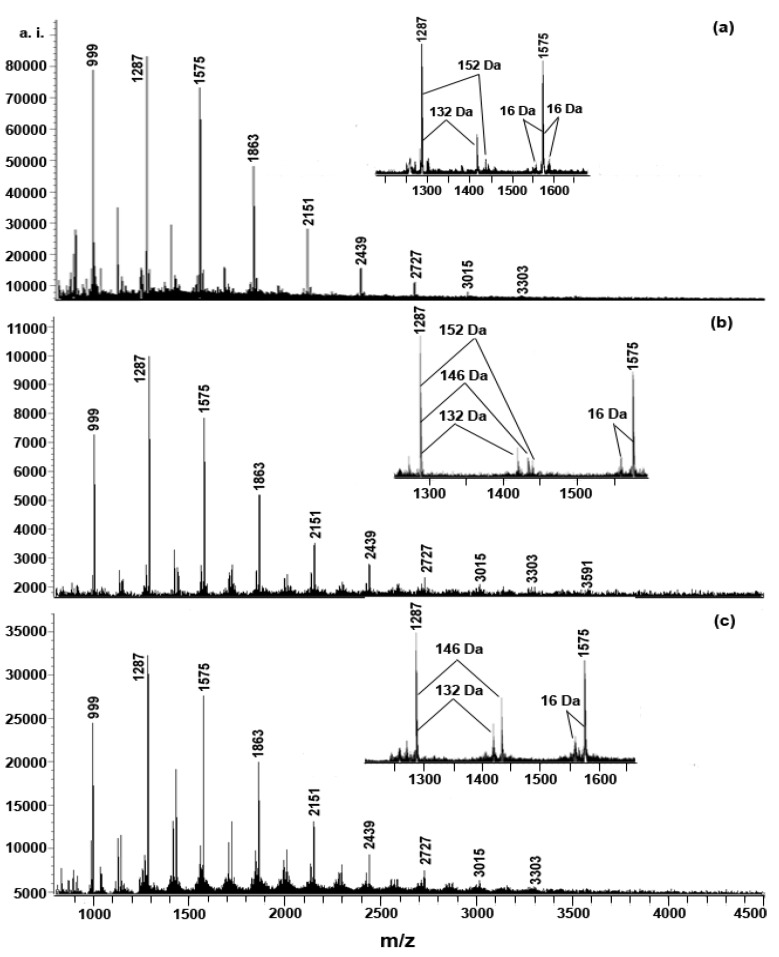
MALDI-TOF positive reflectron mode mass spectra of the condensed tannins from different parts of *A. confusa*: (a) leaf, (b) stem bark, (c) root bark.

Figure 2 shows the MALDI-TOF mass spectra of the polymeric tannin mixture s of the different parts of *A. confusa*, recorded as Cs^+^ adducts in the positive ion reflectron mode. The polymeric character is reflected by the periodic peak series representing different polymers. Condensed tannins isolated from the leaf, stem bark and root bark are characterized by mass spectra with a series of peaks with distances of 288 Da, corresponding to one catechin/epicatechin monomer, therefore, prolongation of condensed tannins is due to the addition of catechin/epicatechin monomers (Table 2).

**Table 2:** MALDI-TOF MS of condensed tannins from different parts of *A. confusa*.

Polymer	n_1_	n_2_	n_3_	n_4_	n_5_	Calculated [M + Cs]^+^	Observed [M + Cs]^+ ^		
							Leaf	Stem bark	Root bark
Trimer	0	3	0	0	0	999	999	999	999
	1	2	0	0	0	983	983	983	983
	0	2	1	0	0	1015	1015	--	--
	0	3	0	1	0	1145	--	1145	1145
	0	3	0	0	1	1151	1151	1151	--
Tetramer	0	4	0	0	0	1287	1287	1287	1287
	1	3	0	0	0	1271	1271	1271	1271
	0	3	1	0	0	1303	1303	--	--
	0	4	0	1	0	1433	--	1433	1433
	0	4	0	0	1	1439	1439	1439	--
Pentamer	0	5	0	0	0	1575	1575	1575	1575
	1	4	0	0	0	1559	1559	1559	1559
	0	4	1	0	0	1591	1591	--	--
	0	5	0	1	0	1721	--	1721	1721
	0	5	0	0	1	1727	1727	1727	--
Hexamer	0	6	0	0	0	1863	1863	1863	1863
	1	5	0	0	0	1847	1847	1847	1847
	0	5	1	0	0	1879	1879	--	--
	0	6	0	1	0	2009	--	2009	2009
	0	6	0	0	1	2015	2015	2015	--
Heptamer	0	7	0	0	0	2151	2151	2151	2151
	1	6	0	0	0	2135	2135	2135	2135
	0	6	1	0	0	2167	2167	--	--
	0	7	0	1	0	2297	--	2297	2297
	0	7	0	0	1	2303	2303	2303	--
Octamer	0	8	0	0	0	2439	2439	2439	2439
	1	7	0	0	0	2423	2423	2423	2423
	0	7	1	0	0	2455	2455	--	--
	0	8	0	1	0	2585	--	2585	2586
	0	8	0	0	1	2591	2591	2591	--
Nonamer	0	9	0	0	0	2727	2727	2727	2727
	1	8	0	0	0	2711	--	2711	2711
	0	9	0	0	1	2873	--	2873	--
Decamer	0	10	0	0	0	3015	3015	3015	3015
	0	10	0	1	0	3161	--	3161	3161
Undecamer	0	11	0	0	0	3303	3303	3303	3303
Dodecamer	0	12	0	0	0	3591	--	3591	--

n_1:_ Number of afzelechin/epiafzelechin units; n_2_: Number of catechin/epicatechin units; n_3_: Number of gallocatechin/epigallocatechin units; n_4_: Number of rhamnoside; n_5_: Number of galloyl units; “--” means no observed peaks corresponding to the calculated ones.

The condensed tannins from the three different parts of *A. confusa* had different polymer chain length varying from trimers to undecamers for leaf and root bark and to dodecamers for stem bark. The spectra did not contain ions with 2 Da lower than that of the highest peaks among the polyflavan-3-ols polymers. In addition to the predicted homopolyflavan-3-ol mass series mentioned above, each DP had a subset of masses 16 Da lower in the spectra of stem bark and root bark (Figure 2 and Table 2). These masses indicated the polymer chains containing monomers with only one hydroxyl group (16 Da) on the aromatic ring B. In contrast, the mass spectrum of leaf condensed tannins is more complicated. Each DP had a subset of masses 16 Da lower, and the subset of masses 16 Da higher were also detected, which can be explained by heteropolymers of repeating flavan-3-ol units containing an additional hydroxyl group at the position 5' of the B-ring. Given the absolute masses corresponding to each peak, it was further suggested that the condensed tannins from stem bark and root bark contain propelargonidin and procyanidin, and the leaf condensed tannins contain propelargonidin, procyanidin and prodelphinidin, all with the procyanidin dominating.

Each peak of the condensed tannins was also followed by mass signals at a distance of 152 Da (corresponding to the addition of one galloyl group at the heterocyclic C-ring) in spectra of leaf and stem bark, 146 Da (corresponding to the addition of one rhamnoside group at the heterocyclic C-ring) in spectra of stem bark and root bark, and 132 Da (corresponding to the addition of one arabinoside group at the heterocyclic C-ring or additional one CS^+^) in spectra of leaf, stem bark and root bark. No series of compounds that are 2 Da multiples lower than those described peaks for heteropolyflavan-3-ols were detected, so A-type interflavan ether linkage does not occur between adjacent flavan-3-ol subunits for leaf, stem bark and root bark. All compounds are linked by B-type bonds. Structures of condensed tannins from different parts of *A. confusa* were thus successfully characterized using MALDI-TOF MS for the first time.

### 2.3. DPPH radical scavenging activity

The 1,1-diphenyl-2-picrylhydrazyl (DPPH) radical is usually used as a reagent to evaluate the free radical scavenging activity of antioxidants [35]. DPPH is a stable free radical and accepts an electron or hydrogen radical to become a stable diamagnetic molecule [36].

**Figure 3:**
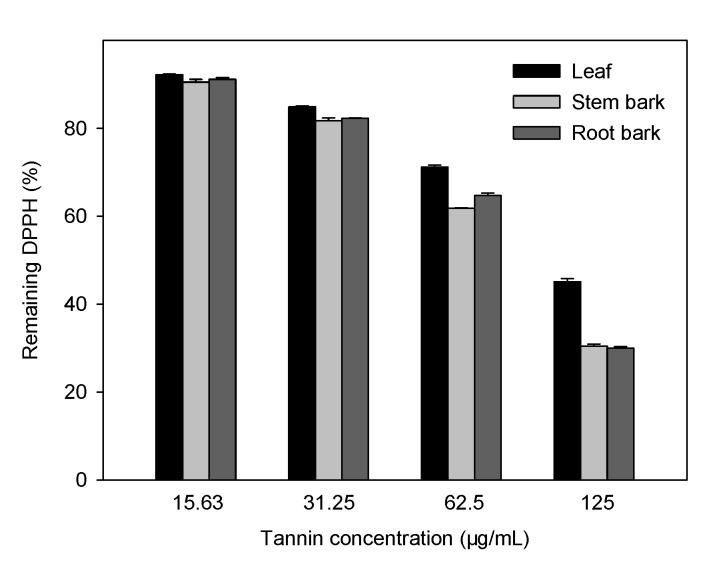
Remaining DPPH after addition of the condensed tannins from different parts of *A. confusa* for 30 min.

The reduction capability of DPPH radical is determined by the decrease in absorbance at 517 nm induced by antioxidants [37]. The percentages of DPPH remaining in the presence of condensed tannins from different parts of *A. confusa* at different concentrations are shown in Figure 3. A dose-response relationship is found in the radical scavenging activity; the activity increased with the increasing concentration of condensed tannins. The quality of the antioxidants about the condensed tannins from different parts of *A. confusa* was determined by the IC_50_ values (the concentration with scavenging activity of 50%). A lower value of IC_50_ indicates greater antioxidant activity.

The IC_50_ values of the stem bark (87.85 ± 0.52 µg/mL) and root bark (89.03 ± 0.50 µg/mL) were significantly lower than those of leaf and other two standards (ascorbic acid and BHA), indicating the condensed tannins of stem bark and root bark exhibited the higher radical scavenging effect than them. The scavenging effect on the DPPH radical decreased in the order: stem bark ≈ root bark > leaf > ascorbic acid > BHA.

**Table 3:** Antioxidant activities of the condensed tannins from different parts of *A. confusa* using the (DPPH) free radical scavenging assay and the (FRAP) ferric reducing antioxidant assay.

Samples	Antioxidant activity	
	IC_50/DPPH_ (µg/mL) *^a^*	FRAP (mmol AAE/g) *^b^*
Leaf	113.06 ± 1.52c	5.92 ± 0.04a
Stem bark	87.85 ± 0.52d	5.89 ± 0.14a
Root bark	89.03 ± 0.50d	5.69 ± 0.09a
Ascorbic acid	118.88 ± 3.33b	--
BHA	126.21 ± 1.32a	4.93 ± 0.09b

*^a^* The antioxidant activity was evaluated as the content of the test sample required to decrease the absorbance at 517 nm by 50% in comparison to the control; *^b^* FRAP values are expressed in mmol ascorbic acid equivalent/g sample in dry weight; BHA: Butylated hydroxyanisole. Values are expressed as mean of duplicate determinations ± standard deviation; Different letters in the same column show significant differences from each other at *P* < 0.05 level.

### 2.4. Ferric reducing antioxidant power (FRAP)

**Figure 4:**
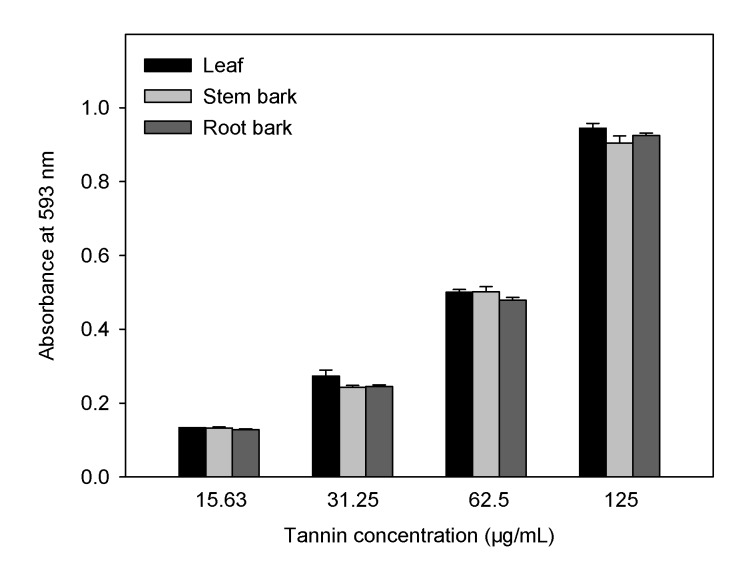
Ferric reducing power after addition of the condensed tannins from different parts of *A. confusa.*

The FRAP assay is based on the redox reaction of ferric ion in the presence of a reducer. The reduction capacity of a compound may serve as a significant indicator of its potential antioxidant activity [38]. A higher absorbance corresponds to a higher ferric reducing power. All tannins showed increased ferric reducing power with the increasing concentration (Figure 4). At 125 µg/mL, the reducing power of leaf (*A_593_* = 0.94 ± 0.01) was superior to stem bark (*A_593_* = 0.90 ± 0.02). The FRAP value, used to determine the antioxidant ability of different parts of *A. confusa* in present study, was expressed in ascorbic acid equivalents. The FRAP values for leaf, stem bark and root bark ranged from 5.69 ± 0.09 to 5.92 ± 0.04 mmol AAE/g dried tannins, and were all significantly higher than that of BHA (4.93 ± 0.09 mmol AAE/g dried sample).

## 3. Experimental

### 3.1. Chemicals and materials

All solvents used were of analytical reagent (AR) purity grade. 1,1-Diphenyl-2-picrylhydrazyl (DPPH), 2,4,6-tripyridyl-*S*-triazine (TPTZ), ascorbic acid, butylated hydroxyanisole (BHA), and cesium chloride were purchased from Sigma-Aldrich (USA). Sephadex LH-20 was purchased from Amersham (USA). Leaf, stem bark and root bark of *A. confusa* were collected from Xiamen Botanical Garden, Fujian Province, China.

### 3.2. Extraction and purification of the condensed tannins

Freeze-dried leaf, stem bark and root bark powders (35 g of each) were extracted thrice with 7:3 (v/v) acetone-water solution (3 × 250 mL) at room temperature. Each extract was filtered and pooled, and the solvent was removed under reduced pressure by use a rotary evaporator at 38 °C. The remaining aqueous fraction was extracted thrice with hexane (3 × 150 mL) in order to remove chlorophyll and lipophilic compounds. The remaining crude tannin fraction was chromatographed on an LH-20 column (Pharmacia Biotech, Uppsala, Sweden) which was first eluted with methanol-water (50:50, v/v) and then with acetone-water (7:3, v/v). The last fraction of purified condensed tannins was freezed-dried and stored at -20°C before analysis by MALDI-TOF mass spectrometry.

### 3.3. Determination of total phenolics and extractable condensed tannins

Established procedures [39] were used. Total phenolic content was determined by the Prussian blue method [40]. Extractable condensed tannin content was assayed by the butanol-HCl method [41]. All used respective purified condensed tannins as the standards.

### 3.4. MALDI-TOF MS analysis

The MALDI-TOF MS spectra were recorded on a Bruker Reflex III instrument (Germany). The irradiation source was a pulsed nitrogen laser with a wavelength of 337 nm, and the duration of the laser pulse was 3 ns. In the positive reflectron mode, an accelerating voltage of 20.0 kV and a reflectron voltage of 23.0 kV were used. 2,5-Dihydroxybenzoic acid (DHB, 10 mg/mL 30% acetone solution) was used as the matrix. The sample solutions (10 mg/mL 30% acetone solution) were mixed with the matrix solution at a volumetric ratio of 1:3. The mixture (1 µL) was spotted to the steel target. Amberlite IRP-64 cation-exchange resin (Sigma-Aldrich, USA), equilibrated in deionized water, was used to deionize the analyte-matrix solution thrice. Cesium chloride (1.52 mg/mL) was mixed with the analyte-matrix solution (1:3, v/v) to promote the formation of a single type of ion adduct ([M+Cs]^+^) [42].

### 3.5. DPPH radical scavenging activity

The effect of purified condensed tannins on DPPH radical was determined according to the method of Braca *et al*. [43]. Aliquots (0.1 mL) of various concentrations of each freeze-dried sample at different concentrations (15.63–125 µg/mL) was added to DPPH solution (3 mL, 0.1 mM in methanol solution). An equal amount of methanol and DPPH served as control. After the mixture was shaken and left temperature for 30 min, the absorbance at 517 nm was measured. Lower absorbance of the reaction mixture indicates higher free radical scavenging activity. The IC_50_ value, defined as the amount of antioxidant necessary to decrease the initial DPPH concentration by 50%, was calculated from the results and used for comparison. The capability to scavenge the DPPH radical was calculated by using the following equation:

DPPH scavenging effect (%) = [(A_1_–A_2_)/A_1_] ×100

where A_1_ = the absorbance of the control reaction; A_2_ = the absorbance in the presence of the sample. BHA and ascorbic acid were used as standards.

### 3.6. Ferric reducing/antioxidant power (FRAP) assay

FRAP assay is a simple and reliable colorimetric method commonly used for measuring the total antioxidant capacity [44]. In brief, prepared freshly FRAP reagent (3 mL) was mixed with test sample (0.1 mL) or methanol (for the reagent blank, 0.1 mL). The FRAP reagent was prepared from 300 mmol/L acetate buffer (pH 3.6), 20 mmol/L ferric chloride and 10 mmol/L TPTZ made up in 40 mmol/L hydrochloric acid. All the above three solutions were mixed together in the ratio of 25:2.5:2.5 (v/v/v). The absorbance of reaction mixture at 593 nm was measured spectrophotometrically after incubation at 25 °C for 10 min. The FRAP values, expressed in mmol ascorbic acid equivalents (AAE)/g dried tannins, were derived from a standard curve.

### 3.7. Statistical analysis

All data were expressed as means ± standard deviation of three independent determinations. One-way analysis of variance (ANOVA) was used, and the differences were considered to be significant at *P* < 0.05. All statistical analyses were performed with SPSS 13.0 for Windows.

## 4. Conclusions

Structures of condensed tannins from leaf, stem bark and root bark of *A. confusa* were characterized by MALDI-TOF MS analysis that showed that the condensed tannins from stem bark and root bark contain propelargonidin and procyanidin, and the leaf condensed tannins contain propelargonidin, procyanidin and prodelphinidin, all with the procyanidin dominating. The condensed tannins had different polymer chain lengths, varying from trimers to undecamers for leaf and root bark and to dodecamers for stem bark. The condensed tannins extracted from the leaf, stem bark and root bark all showed very good DPPH radical scavenging activity and ferric reducing power, suggesting that these extracts may be considered as new sources of natural antioxidants for food and nutraceutical products.
